# Attention-based 3D convolutional recurrent neural network model for multimodal emotion recognition

**DOI:** 10.3389/fnins.2023.1330077

**Published:** 2024-01-10

**Authors:** Yiming Du, Penghai Li, Longlong Cheng, Xuanwei Zhang, Mingji Li, Fengzhou Li

**Affiliations:** ^1^School of Integrated Circuit Science and Engineering, Tianjin University of Technology, Tianjin, China; ^2^China Electronics Cloud Brain (Tianjin) Technology Co, Ltd., Tianjin, China; ^3^School of Information Engineering, China University of Geosciences, Beijing, China

**Keywords:** electroencephalogram (EEG), emotion recognition, attention mechanism, convolutional neural network (CNN), 3D feature construction module, multimodal recognition

## Abstract

**Introduction:**

Multimodal emotion recognition has become a hot topic in human-computer interaction and intelligent healthcare fields. However, combining information from different human different modalities for emotion computation is still challenging.

**Methods:**

In this paper, we propose a three-dimensional convolutional recurrent neural network model (referred to as 3FACRNN network) based on multimodal fusion and attention mechanism. The 3FACRNN network model consists of a visual network and an EEG network. The visual network is composed of a cascaded convolutional neural network–time convolutional network (CNN-TCN). In the EEG network, the 3D feature building module was added to integrate band information, spatial information and temporal information of the EEG signal, and the band attention and self-attention modules were added to the convolutional recurrent neural network (CRNN). The former explores the effect of different frequency bands on network recognition performance, while the latter is to obtain the intrinsic similarity of different EEG samples.

**Results:**

To investigate the effect of different frequency bands on the experiment, we obtained the average attention mask for all subjects in different frequency bands. The distribution of the attention masks across the different frequency bands suggests that signals more relevant to human emotions may be active in the high frequency bands γ (31–50 Hz). Finally, we try to use the multi-task loss function Lc to force the approximation of the intermediate feature vectors of the visual and EEG modalities, with the aim of using the knowledge of the visual modalities to improve the performance of the EEG network model. The mean recognition accuracy and standard deviation of the proposed method on the two multimodal sentiment datasets DEAP and MAHNOB-HCI (arousal, valence) were 96.75 ± 1.75, 96.86 ± 1.33; 97.55 ± 1.51, 98.37 ± 1.07, better than those of the state-of-the-art multimodal recognition approaches.

**Discussion:**

The experimental results show that starting from the multimodal information, the facial video frames and electroencephalogram (EEG) signals of the subjects are used as inputs to the emotion recognition network, which can enhance the stability of the emotion network and improve the recognition accuracy of the emotion network. In addition, in future work, we will try to utilize sparse matrix methods and deep convolutional networks to improve the performance of multimodal emotion networks.

## 1 Introduction

Emotion recognition and analysis are crucial in our everyday lives, particularly in the fields of human-computer interaction (Liu Y. et al., 2020; Cheng et al., 2021), the assessment of psychological disorders such as depression and autism (Blankertz et al., 2016), and fatigue driving (Kong et al., 2017). There are two distinct categories of emotional recognition signals: physiological and non-physiological. Electromyography (EMG), electroencephalography (EEG), electrocardiogram, heart rate (Doma and Pirouz, 2020) and respiratory rate are examples of physiological signals, while facial expressions, utterances, and body postures are examples of non-physiological signals (Daros et al., 2013; Huang et al., 2020).

EEG is noninvasive, practical, quick, and affordable. Consequently, it is frequently employed to examine the brain’s response to emotional stimuli. We can acquire emotion-related feature information from different frequency bands and electrodes of the EEG, and use deep learning methods for feature learning and classification. Wang et al. (2018) used a 3D CNN network to extract spatial features from EEG signals followed by emotion state prediction, but did not consider the effect of temporal feature components in EEG signals on emotion recognition; Yang et al. (2018a) extracted spatio-temporal feature information from EEG signals by cascading CNN and LSTM networks, which is similar to the emotion recognition architecture based on convolutional recurrent networks proposed in this paper, but the method proposed by Yang et al. did not integrate feature information of EEG data in different dimensions, which resulted in spatio-temporal features representativeness extracted by the CNN-LSTM network did not comprehensive; Li et al. (2018) constructed a two-dimensional matrix of 62 electrode locations and mapped the EEG features onto the two-dimensional matrix, they were then fed into a network model for training; Song et al. designed differential entropy features based on the relationship between electrode locations and used a graph convolutional neural network as a classifier (Song et al., 2020); both Li et al. and Song et al. only considered the effect of relative position information between different electrodes on emotion recognition, ignoring the importance of information from different frequency bands within the same electrode for the prediction of emotional states; Yang et al. used a combination of four frequency bands in the EEG, including theta (4–7 Hz), α (8–13 Hz), β (14–30 Hz), and γ (31–50 Hz), and found that they are closely related to emotional states (Yang et al., 2018b), but did not use attentional means to adjust the weight parameters of the different frequency bands according to their importance to help the emotion network to better fulfill the emotion prediction task. To address the weaknesses and shortcomings of the above research methods, in this paper, we propose a three-dimensional convolutional recurrent neural network model based on the attention mechanism, 3FACRNN, which can first integrate the multidimensional feature information of EEG signals using the three-dimensional feature construction module to increase the feature complexity of EEG signals, then extract the deep spatio-temporal features of EEG signals using the convolutional recurrent neural network, and finally combine with the frequency-band attention module and the self-attention module to improve the discriminative capability of the feature information.

Inspired by the research of Shen et al. (2020), this paper proposes a 3D feature construction module to better utilize all the emotional information contained in the EEG signals. This 3D feature building module can extract frequency band, spatial and temporal information from the original EEG signals, and then input the 3D-structured EEG signals into a neural network consisting of CNNs and LSTMs for deeper feature abstraction, and finally input them into a SoftMax classifier for emotional state classification. Incorporating attentional mechanisms, such as frequency bands and self-attention mechanisms into this procedure allows us to extract more discriminative feature information (Guo et al., 2022; Tao et al., 2023). Although all four bands of EEG signals contain information related to emotions, the importance of the emotional information contained in different bands varies. To deal with this case, we used 1×1 convolution method to assign different weights to different bands. In addition, since the importance of different EEG samples of subjects varies, we integrate a self-attention module in LSTM, which can extract the attention information of subjects according to the importance of their different EEG samples. Through the attention mechanism, the 3FACRNN network is able to acquire more discriminative feature information from EEG signals, thereby enhancing its recognition performance.

The EEG signal is the result of the integrated activity of human brain regions, and because it is not influenced by subjective human factors, it accurately reflects the true emotional state of a person in response to a stimulus. However, the EEG signal is easily disturbed by noise. Although facial expression can visually communicate the subject’s emotional state, it is often disguised, so the subject is sometimes unable to express his or her own emotional state accurately. Multimodal emotion recognition methods that combine the facial expressions of subjects with EEG signals can compensate for the deficiencies of unimodal methods and achieve superior recognition results (Mühl et al., 2014; D’mello and Kory, 2015; Basbrain and Gan, 2020). Afouras et al. (2020) trained a visual recognition model based on lip reading using the knowledge of obscurity in the speech modality, but both the speech and visual modalities are artifactual and do not allow for true emotional state labeling. Soleymani et al. (2016) proposed a multimodal continuous emotion prediction method based on facial sign sequences and EEG signals, obtaining high recognition accuracy. Tzirakis et al. (2017) achieved the first end-to-end emotion recognition by using ResNet and two convolutional layers to extract facial expression feature signals and speech feature signals, concatenating them to form new features, and then integrating contextual information via a multilayer LSTM. Both the studies of Soleymani et al. and Tziraki et al. fused the feature information of the two modalities in series at the feature level or decision level, without considering the differences and complementarities between the features. In contrast, the multimodal emotional network 3FACRNN network proposed in this paper does not serially splice features between two modalities and feed them into the network for undifferentiated learning, but rather forces approximation of the intermediate feature vectors of the visual and EEG modalities through the multitasking loss function Lc, with the aim of utilizing the knowledge of the visual modalities for the improvement of the performance of the EEG network model.

In this paper, we propose a novel multimodal emotion recognition network (3FACRNN) based on the attention mechanism, which includes visual and EEG networks. The visual network is trained only for the visual modality, and the important feature information in the visual modality is extracted and used to supervise the EEG network for training and learning. The proposed multi-task loss function L_C_ consists of the weighted sum of the L_1_ loss function and the cross-entropy loss function, which is used to force the approximation of the intermediate feature vectors of the two modalities, so that the EEG modalities can learn the knowledge of the visual modalities, thereby improving the recognition performance of the EEG network. The EEG network model cascades the 3D feature construction module, the multi-attention module, the CNN, and the LSTM framework, The inputs to the EEG network include intermediate feature vectors obtained from the visual network and raw EEG signals and labels. The raw EEG signals are first preprocessed to remove noise, artifacts, and baseline signals, and then important feature information is integrated into the EEG signals using a 3D feature building module, and then important band information and intrinsic similarity information of different EEG signals are extracted using a multi-attention module, and finally, a convolutional recurrent neural network to extract local features for deeper feature abstraction, and a classifier consisting of a fully connected layer and SoftMax is used to complete the prediction of emotion labels. The proposed 3FACRNN network model for emotion recognition has been evaluated on two publicly available datasets, the DEAP dataset (Koelstra et al., 2012) and the MAHNOB-HCI dataset (Soleymani et al., 2012). On both datasets, the network model has demonstrated outstanding recognition accuracy. Here is a synopsis of our most notable contributions:

1. This paper proposes a 3D convolutional recurrent neural network model based on the attention mechanism called 3FACRNN, which cascades a 3D feature construction module, a frequency band attention module, a convolutional recurrent neural network, and a self-attention module to perform the emotion recognition task. This model can effectively enhance the discriminative properties of EEG signals in space, time, and spectrum.
2. In this paper, we use the multi-task loss function Lc to force approximation of the intermediate feature vectors of visual modality and EEG modality in order to achieve the purpose of using the dark knowledge of visual modality to supervise the emotion recognition of the EEG network, which effectively utilizes the advantage of the high resolution of the visual modality in spatial dimensions, solves the problem of the single data information in the uni-modal approach, and improves the feature complexity of the EEG signals in spatial dimensions.
3. The average accuracy and standard deviation of the proposed 3FACRNN model on the valence and arousal dimensions of the DEAP and MAHNOB-HCI datasets were 96.75 ± 1.75, 96.86 ± 1.33, 97.55 ± 1.51, and 98.37 ± 1.07. It outperforms existing emotion recognition methods using multimodal data. Moreover, this paper analyses the attentional weights of various frequency bands, and the weight distribution suggests that the gamma band of EEG signals may be more pertinent to human emotions.

The remaining sections of the paper are organized as follows: section 2 describes the relevant materials and methodologies, section 3 analyses the experimental results, section 4 discusses the work accomplished and concludes the entire paper.

## 2 Methods

**Figure 1:**
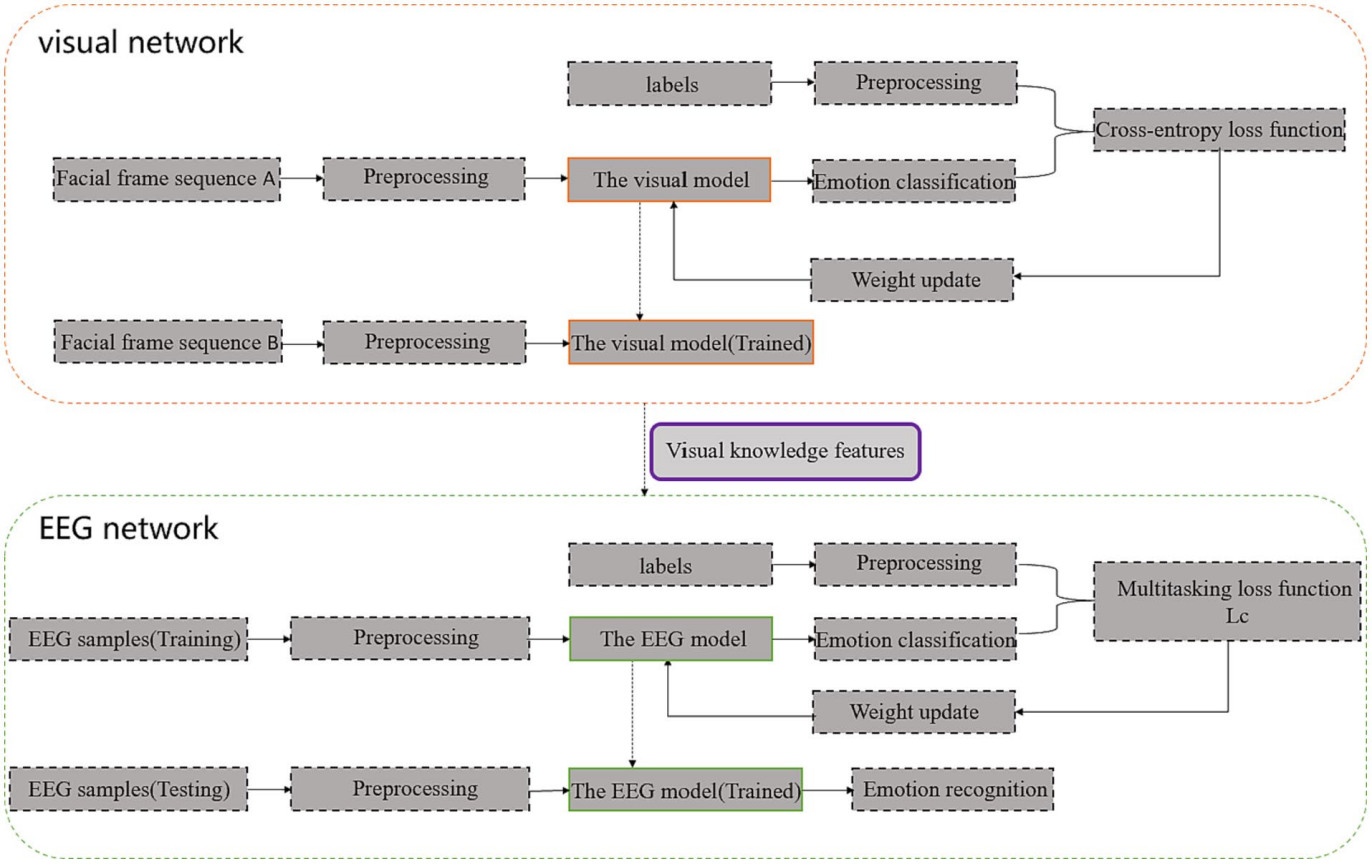
3FACRNN network architecture (Facial frame sequence A is obtained after preprocessing of facial expression videos in the AFEW-VA dataset, and f Facial frame sequence B is obtained after preprocessing of facial expression videos in the DEAP dataset and MAHNOB-HCI dataset).

The framework of the proposed 3FACRNN multimodal emotional network model is shown in Figure 1. It is made up of the visual and EEG networks.

The facial video frames of the subjects in the DEAP dataset and the MAHNOB-HCI dataset were then fed into the pre-trained visual network to extract the spatio-temporal eigenvectors of the visual modalities. The feature information obtained from the visual modalities along with the original EEG signal and labels was fed into the EEG network so that the dark knowledge of the visual modalities could enhance the recognition performance of the EEG network. The specific implementation process of each component in the two subnetworks and the interaction mechanism between the two subnetworks for learning will be described in detail in the following section.

### 2.1 The visual network model

**Figure 2:**
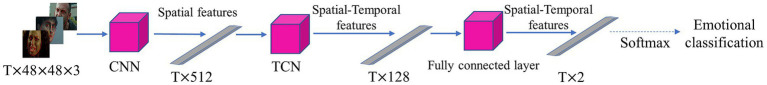
Framework for the visual model.

The visual network model is shown in Figure 2. The visual network model consists of facial video frame acquisition, pre-processing, CNN, time series convolutional network (TCN) and SoftMax classifier architecture. The facial expression is first processed by frame extraction, and the processed face video frames are resized to 48×48 ×3, with T video frames input at a time. It is then passed through spatial–temporal convolutional network, which consists of CNN, TCN and linear layers. The T consecutive video frames and labels are fed into a CNN, which contains two convolutional layers, two pooling layers and a flatten layer. The spatial information of the video frames is derived by the CNN network and spatial features are generated for each frame, resulting in T×512-dimensional spatial features. The latter is then passed through a temporal convolutional neural network (TCN), from which temporal information is obtained and the spatial–temporal composite features of the video frames are obtained, generating T×128-dimensional spatial–temporal features. TCN networks are capable of extracting features at different time scales and can effectively capture long-term dependencies in time-series data (Xue et al., 2020; He et al., 2022; Wang et al., 2022). The TCN network in the visual network proposed in this paper cascades two temporal convolution modules and a pooling layer, and the internal parameters of both temporal convolution modules can be shared. The temporal convolution module consists of one normal convolutional layer, two dilated convolutional layers, and a residual block, with Relu activation functions and normalization layer added after each convolutional and dilated convolutional layer. The convolution kernel size of the convolutional layer is 3, stride = 1, dilation = 1; the convolution kernel size of the dilated convolutional layer is 3, stride = 1, dilation = 2. The role of the dilated convolutional layer is to inject voids into the convolutional layer as a way to increase the receptive field so that the output contains a larger range of feature information than it otherwise would. Where the dilation parameter refers to the number of kernel intervals. A 1×1 convolutional kernel is used in the residual block to perform dimensional matching of the input to the output and to residually connect the input to the output to prevent gradient explosion. In addition we added Dropout layer after each normalization layer for preventing overfitting and its scale is set to 0.5. The T × 128 dimensional spatio-temporal features are then mapped to T × 2 dimensions using a fully connected layer. Finally, the SoftMax classifier receives the extracted features as input to recognize the emotional state., using a cross-entropy loss function to reduce the distance between the predicted sequence and the actual sequence (labels). We used a large number of 2D-based facial image or emotion databases AFEW-VA (Kossaifi et al., 2017) and AffectNet (Mollahosseini et al., 2019) to train the visual network.

### 2.2 The EEG network model

**Figure 3:**
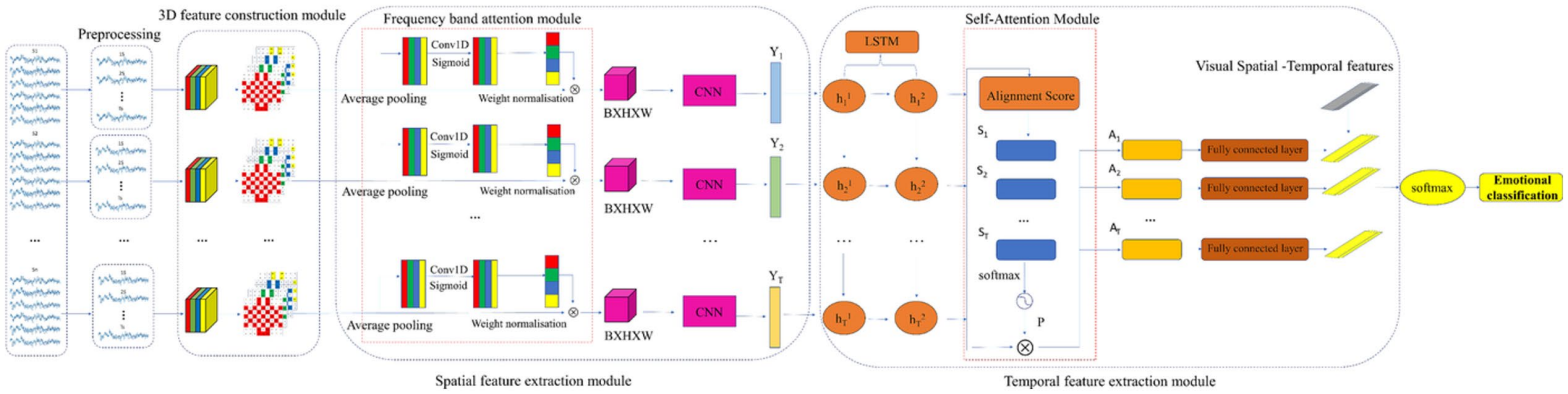
Framework for the EEG model.

Figure 3 depicts the EEG network model, which consists of an EEG signal preprocessing, a 3D feature construction module (feature extraction), a convolutional recurrent neural network (frequency band attention module, CNN, LSTM, self-attention module), and a SoftMax classifier. First, we pre-process the raw EEG signal to eliminate the baseline signal, and then we input the pre-processed EEG signal into the 3D feature construction module to integrate the frequency information, spatial information, and temporal information of the signal. The 3D EEG signals are then fed into a convolutional recurrent neural network. The frequency band attention module in the convolutional recurrent neural network captures the frequency bands that are more critical to the task. The self-attention mechanism focuses on more important EEG samples by assessing the probability based on the similarities between samples. The CNN and LSTM networks further abstract and extract the spatial–temporal features of the EEG signals. Finally, a SoftMax classifier is utilized to predict the subject’s emotional state. Each component’s implementation is described in detail below.

#### 2.2.1 3D feature construction module

Initially, we carry out preprocessing procedures on the unprocessed EEG signals, which encompass both baseline and experimental signals (Ahmed et al., 2023). In this paper, we use a non-overlapping sliding window to remove the baseline signal from the raw EEG signal. According to previous studies, human emotional state is generally maintained between 1 and 12 s, while 0.5–3 s can achieve better classification accuracy (Li et al., 2017). For the DEAP dataset, each subject has 40 × 60 s of emotional EEG signals, and we set the size of the sliding window to 2 s without overlapping, so that a total of 26,400 samples can be obtained, with 1,200 samples for each person; while for the MAHNOB-HCI dataset, due to the varying durations of each trial, we choose the middle 30 s of each video as the experimental video data, and set the size of the sliding window to 0.5 s without overlapping, so that a total of 30,000 samples can be obtained, with 1,200 samples for each person.

The pre-processed EEG signals were fed into the 3D feature construction module. Each time-slice sample was first decomposed into four frequency bands, i.e., θ (4–7 Hz), α (8–13 Hz), β (14–30 Hz) and γ (31–50 Hz), using a Butterworth filter (Zheng and Lu, 2015), and then the differential entropy (DE) features of these four bands were calculated separately. The researchers found that the differential entropy feature is currently the most effective feature in the field of emotion recognition (Chen et al., 2019), and the formula is shown in Eq. (1).


(1) $$D\left(x\right)=-\int_{x}f\left(x\right)\text{logf}\left(x\right)dx$$

where $f\left(x\right)$ is the probability density function of x. According to Zheng et al. the differential entropy characteristic formula for the Gaussian distribution is shown in Eq. (2), where e is Euler’s constant and σ is the standard deviation of the EEG sequence.


(2) $$\begin{array}{l} h\left(z\right)=\int_{-\infty }^{+\infty }\frac{1}{\sqrt{2\pi \sigma^{2}}}\exp\frac{{\left(z-u\right)}^{2}}{2\sigma^{2}}\log\frac{1}{\sqrt{2\pi \sigma^{2}}}\\ \;\exp\frac{{\left(z-u\right)}^{2}}{2\sigma^{2}}dz=\frac{1}{2}\log2\pi e\sigma \end{array}$$

**Figure 4:**
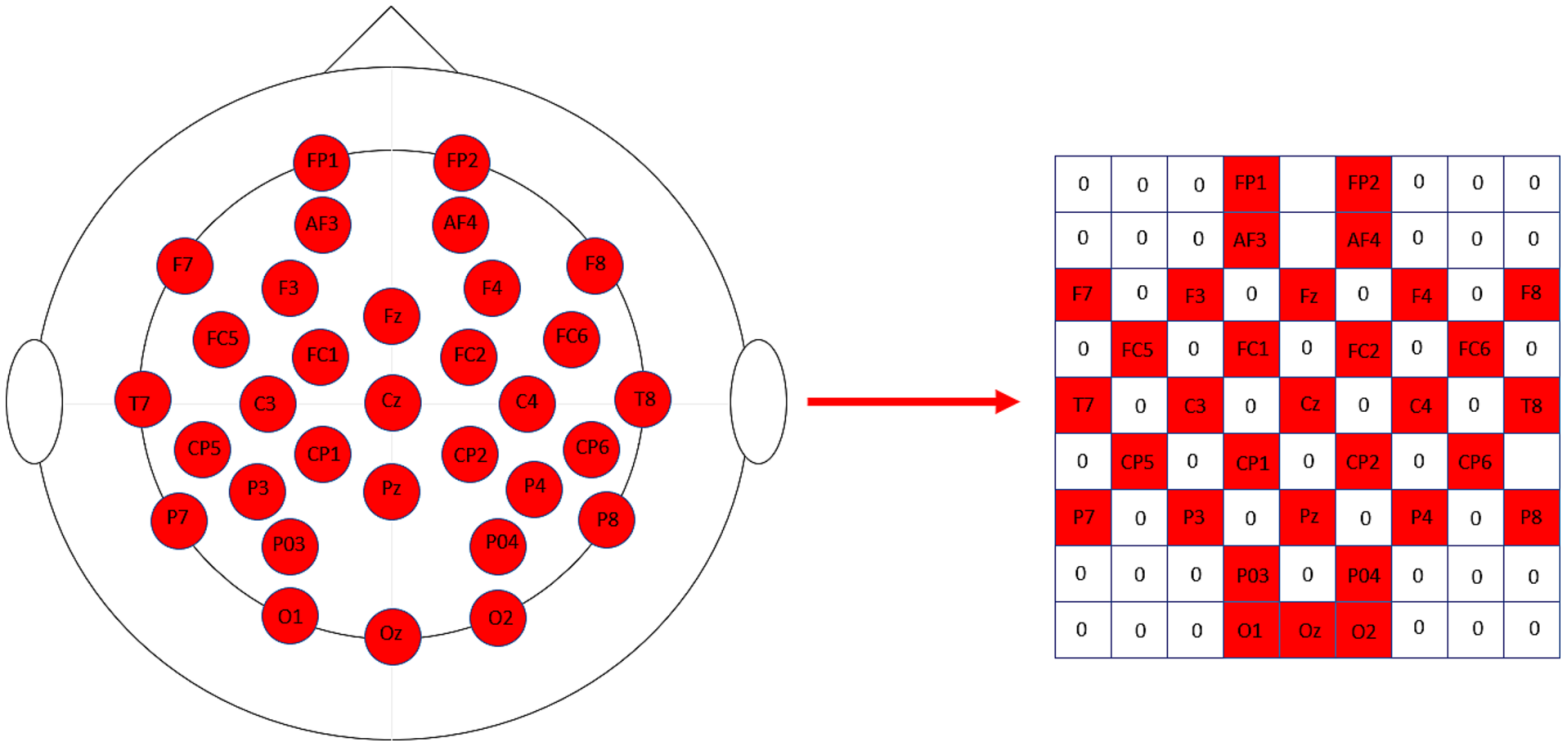
The 32 sampled electrodes are projected onto the 2D map on the right according to their relative positions.

The differential entropy features of each frequency band were then projected onto a two-dimensional matrix (Li et al., 2018; Nguyen et al., 2019; Sha et al., 2023), with the length and width of the two-dimensional matrix set to H = 9 and W = 9, respectively, and the relative positions of the actual recording electrodes corresponding to the positions of the recording electrodes in the two-dimensional matrix. Figure 4 shows the two-dimensional matrix obtained from the projection based on 32 sampled electrodes, with the unused channel signals filled with zeros. Finally, the four frequency bands of each EEG sample were stacked to obtain a three-dimensional feature representation of each EEG signal and as shown in Eq. (3):


(3) $$E_{i}=\left[E_{1},E_{2},E_{3}\ldots E_{T}\right]\in R^{T\times B\times H\times W}$$

#### 2.2.2 Frequency band attention module

The band attention module used in this paper is inspired by the ECAnet Convolutional Attention Module (Han et al., 2021) in the field of image recognition, and uses a one-dimensional convolution to interact with the information in each band, with the size of the convolution kernel varied by an adaptive function. Specifically, for the EEG sample $E_{i}\;\in$*R^B × 9 × 9^*, the matrix with feature maps [B,H,W] is first converted to a vector of [1,c] by a global average pooling layer, and then the one-dimensional convolution kernel size kernel_size is obtained by an adaptive function, the formula of which is shown in Eq. (4):


(4) $$k=\left|\frac{\log\left(c\right)}{y}+\frac{b}{y}\right|$$

Where y*=2* and b*=1*, We calculate the size of kernel_size and apply it to the one-dimensional convolution, then multiply to [1,c] reshape into [c,1] and multiply by with the one-dimensional convolution to get the weight for each band in the feature map, and finally normalize the weights and multiply with by the original feature map to get the weighted feature map and as shown in Eq. (5):


(5) $$E_{i}^{*}=E_{i}⊗\text{Sigmoid}\left(\mathrm{cov}1D(\text{filters}=1,\text{Kenel}:\text{size}=c\right)\left(x\right)$$

#### 2.2.3 The CNN-LSTM networks

The CNN-LSTM networks consists of four successive convolutional layers, a maximum pooling layer, a fully connected layer and an LSTM layer. There are four successive convolutional layers with convolutional kernel sizes of 5×5, 4×4, 3×3 and 1×1, and output channels of 64, 128, 256 and 128, in respectively, all of which apply the zero-filling and RELU activation functions are applied. A maximum pooling layer with convolutional kernel size 2×2 and step size 2 is used to improve the robustness of the network, the output of the pooling layer is flattened and fed into the fully connected layer, which outputs 512 units, and Et is set as the input of the CNN, $E_{t}\;\in$ *R^1 × B × H × W^*, and Eqs. (6)–(11) are used to describe the computation of the layers in the CNN:


(6) $$C_{1}=f\left(\text{Conv}\left(E_{t_{i}},w_{c_{1}}\right)\right),W_{c_{1}}\in R^{5\times 5}$$

(7) $$C_{2}=f\left(\text{Conv}\left(C_{1},w_{c_{2}}\right)\right),W_{c_{2}}\in R^{4\times 4}$$

(8) $$C_{3}=f\left(\text{Conv}\left(C_{2},w_{c_{3}}\right)\right),W_{c_{3}}\in R^{3\times 3}$$

(9) $$C_{4}=f\left(\text{Conv}\left(C_{3},w_{c_{4}}\right)\right),W_{c_{4}}\in R^{1\times 1}$$

(10) $$C_{5}=f\left(\text{Conv}\left(C_{4},w_{c_{5}}\right)\right),W_{c_{5}}\in R^{2\times 2}$$

(11) $$y_{t}=\text{linea}\left(\text{flatten}\left(C_{5}\right)\right)$$

Where $f\left(\text{.}\right)$ denotes the Relu activation function, $W_{c1}$, $W_{c2}$, $W_{c3}$, $W_{c4}$, $W_{c5}$ denote the convolution kernel of each convolutional layer, and $E_{t}$ is the input matrix, and we input $E_{t}$ into the spatial convolutional network to obtain the spatial feature representation of $E_{t}$, $y_{t}$. where, $y_{t}$ t = 1,2,…T denotes the feature vector from the 1st sample, 2nd sample to the Tth sample.y* is obtained by concatenating all the feature vectors y_t_ in chronological order.

The CNN output sequence is y* = (y_1_, y_2_, y_3_ … y_t_, where $y_{t}\;\in$*R^1 × 512^*, t = 1,2,3. $y_{t}$, where $y_{t}\;\in$*R^1 × 512^*, t = 1,2,3… T), y* is input to the LSTM layer, the number of LSTM layers is set to 2, and the number of hidden units is set to the sample number, so it can be considered that the output of each time step is the spatial–temporal feature information of each sample, and the output of the LSTM network is computed as shown in Eqs. (12)–(16):


(12) $$i_{t}=\sigma \left(W_{yi}y_{t}+W_{hi}h_{t-1}+W_{ci}c_{t-1+}b_{i}\right)$$

(13) $$f_{t}=\sigma \left(W_{yf}y_{t}+W_{hf}h_{t-1}+W_{cf}c_{t-1+}b_{f}\right)$$

(14) $$c_{t}=f_{t}c_{t-1}+i_{t}\tanh\left(W_{yc}y_{t}+W_{hc}h_{t-1}+b_{c}\right)$$

(15) $$o_{t}=\sigma \left(W_{yo}y_{t}+W_{ho}h_{t-1}+W_{co}c_{t+}b_{o}\right)$$

(16) $$h_{t}=o_{t}\tanh\left(c_{t}\right)$$

where σ is the logical sigmoid activation function, i, f, and o are the input, forgetting, and output gates, respectively, and C is the cell activation vector. ht. denotes the Tth output hidden state of the seconds recursive layer and its expression is shown in Eq. (17):


(17) $$h_{t}|h_{t}=\text{Lstm}\left(y_{t}\right),t=1,2,3\ldots T,h_{t}\in R^{T\times 128}$$

#### 2.2.4 Self-attention module

As shown on the right side of Figure 3, the self-attention module aims to assign different weights to each EEG sample in order to explore the importance between different samples and to extract more discriminative spatial–temporal feature information. The feature score vector $S_{t}$ is first computed for each hidden state $h_{t}$ and the formula is shown in Eq. (18):


(18) $$S_{t}=f\left(h_{t},d_{t}\right)=W_{t}\sigma \left(W_{1}h_{t}+W_{2}d_{t}+b_{1}\right)+b_{2}$$

where $f\left(t\right)$ denotes the importance of the Tth coded sample, and $d_{t}$ is the aligned pattern vector generated from $h_{t}$ by linear transformation with the same dimension as $h_{t}$. The activation function is set to Relu, $W_{t}$ and $b_{2}$ denote the weight matrix and bias term of the activation function, respectively. $W_{1}$, $W_{2}$ are the weight matrices of $h_{t}$ and $d_{t}$ and $b_{1}$ is the bias term. The similarity $N_{t}$ within each sample of different points is obtained by dot-multiplying the transpose $S_{t}^{T}$ of the feature score vector with the output hidden state $h_{t}$ The probabilistic representation of $N_{t}$ by SoftMax function, the probability of the Tth hidden layer state can be expressed as shown in Eqs. (19), (20):


(19) $$N_{t}=s_{t}^{T}h_{t}$$

(20) $$P_{t}=\frac{\exp\left(N_{t}\right)}{\Sigma_{t=1}^{T}\exp\left(N_{t}\right)}$$

Each output hidden layer $h_{t}$ is then allowed to multiply with its computed probability to obtain the features extracted by the self-attention module: A = {A_1_,A_2_,A_3_…A_t_}, t = 1,2,3…T. Finally, the extracted spatial–temporal attention features are fed into a classifier consisting of a fully connected layer and a SoftMax layer to output the final emotion type labels.

### 2.3 EEG network enhanced by visual network

We use the knowledge gained from the visual network to improve the performance of the EEG network. Firstly, the facial expression video frames in the emotion database are used to pre-train the visual model, then, the trained visual network is used to extract the features of the visual modality in the target dataset, and finally, the raw EEG signals together with the corresponding labels and features obtained from the visual network are input into the EEG network in an offline manner, and the EEG network is trained using the weighted sum of the cross-entropy function and the L_1_ loss function as the loss function to make the whole The training process is more controllable, and its formula is as shown in Eq. (21):


(21) $$L_{c}=-\rho \sum_{t=1}^{T}Y_{t}\log\left(P_{t}\right)+\left(1-\rho \right)L_{1}\left(V_{t}-V_{s}\right)$$

where $Y_{t}$ denotes the label of the Tth EEG sample, $P_{t}$ denotes the predicted probability of the Tth sample, $V_{t}$ and $V_{s}$ denote the spatial–temporal features of the visual and EEG networks, respectively, and ρ is a hyperparameter that is manually set to 0.8. The cross-entropy loss function is the primary loss function, which updates the weight matrix w in the model by the discrepancy between the actual prediction and the expected label, and reduces the distance between the actual prediction and the expected label, and a lower cross-entropy loss function represents a higher emotion recognition accuracy; the $L_{1}$ loss function is the auxiliary loss function, inspired by Romero et al. (2015), which extracts knowledge by enforcing the proximity of the intermediate features graphs using the $L_{1}$ loss function, and the $L_{1}$ loss function is computed as shown in Eq. (22):


(22) $$L_{1}=w*\frac{1}{TF}\sum_{t=1}^{T}\left|U_{i}-V_{i}\right|$$

where $U_{i}$ ∈ R^T × F^ and $V_{i}$ ∈ R^T × F^ denote the feature sequences obtained at each time step, and $w^{*}$ is the hyperparameter, and the optimal $w^{*}$ is found by grid search. The multi-task loss function $L_{c}$ guides the training process of the EEG network so that the EEG network can learn the Knowledge of the visual network, thus achieving the purpose of improving the emotion recognition performance of the EEG network using the visual modality.

## 3 Results

### 3.1 Introduction to source datasets

To evaluate the efficacy of the proposed network model, we conducted experiments on two multimodal data sets, DEAP and MAHNOB-HCI; Table 1 provides the relevant details for the two datasets.

**Table 1:** Detailed information on the DEAP dataset and the MAHNOB-HCI dataset.

Item	DEAP	MAHNOB-HCI
Subjects	22	25
Trail of each subject	40	20
Each clip duration	60s	30s
Available channels	32	32
Sampling rate	128 Hz	256 Hz
Items for rating emotion	Valence, Arousal	Valence, Arousal

The MAHNOB-HCI is a multimodal emotion database of 30 young healthy adult participants, 17 females and 13 males. Age ranging from 19 to 40 years (M = 26.06 SD = 4.39). The MAHNOB-HCI was used to record responses to emotional stimuli and to record facial expression videos, audio signals, EEG signals and other physiological signals from the 30 participants. After viewing 20 emotional video clips, participants rated their emotional experience on each of the four dimensions of arousal, valence, control and predictability, labeling the dimensions of arousal, valence, control and predictability on a scale of 1–9 on each trial. Facial expression videos were transmitted at 60 frames per second. Due to problems with the experimental equipment or the experimental recording, data were incomplete for five individuals, so the actual number of participants in our experiment was 25, with 20 trials per person for each dimension.

The DEAP dataset is a multimodal dataset for the analysis of human emotional states. EEG and peripheral physiological signals were recorded from 32 participants. 22 of the 32 participants recorded frontal videos using a Sony DCR-HC27E camcorder, so we used the data from these 22 subjects. Subjects watched 40 one-minute music video clips and rated their emotional experience on five dimensions: arousal, valence, dominance, liking and familiarity, on a discrete scale of 1–9, except for familiarity, which was rated on a discrete scale of 1–5. Setting the transmission speed of facial expression videos was set from the original 50 to 60 fps, which is the same as the MAHNOB-HCI dataset, to facilitate subsequent unified processing.

We conducted subject-related emotion recognition experiments on the DEAP dataset and the MAHNOB-HCI dataset to assess the feasibility of the proposed method.

Initially, we examined the efficacy of the 3FACRNN network in identifying emotions using the DEAP dataset and the MAHNOB-HCI dataset. The emotion categories for each trial were hierarchically designated along the dimensions of arousal and valence, respectively, using the subjects’ own levels of arousal and valence from the DEAP and MAHNOB-HCI datasets as the criteria for self-rating emotions. On a scale from 1 to 9, participants rated their arousal and valence, and we chose 5 as the threshold to divide the labels into two binary classification problems. The overall performance of the approach was evaluated by considering the average classification accuracy, precision, recall, and F1 scores across all participants. Next, we performed ablation experiments on the 3FACRNN network to examine the impact of the 3D construction module, attention module, and visual modality on the classification accuracy. Additionally, we calculated the attentional weights of the various bands to assess the significance of each band in the emotion recognition process. Ultimately, we compared the 3FACRNN network model with previously reported methods for the DEAP dataset and the MAHNOB-HCI dataset.

The models used in this paper are implemented by Openface, Keras2.6.0, Keras2.6.0 is extended by Tensorflow2, all model training is performed on NVIDIA GeForce RTX 3060 laptop GPU.

### 3.2 Emotion recognition using 3FACRNN

In order to train the 3FACRNN network model, we initially trained the visual network on the AFEW-VA dataset. This was done as a fine-tuning step for the facial expression recognition task. The learning rate for the visual model was set to 1e-5, the maximum number of epochs was set to 100, and the batch size was set to 128. Additionally, the optimal model parameters were loaded at the end of each epoch. The learning rate for the EEG network model was set to 1e − 6. The maximum number of epochs was set to 100, and the batch size was set to 128. A grid search was performed using Eq. (22), with the parameter w ranging from 0.5 to 1.5 and a step size of 0.1. The hyper-parameters were optimized using the test set. Five times tenfold cross-validation is applied on each subject. The average classification accuracy and standard deviation of all subjects were used as the final results to represent the performance of the 3FACRNN network model.

**Figure 5:**
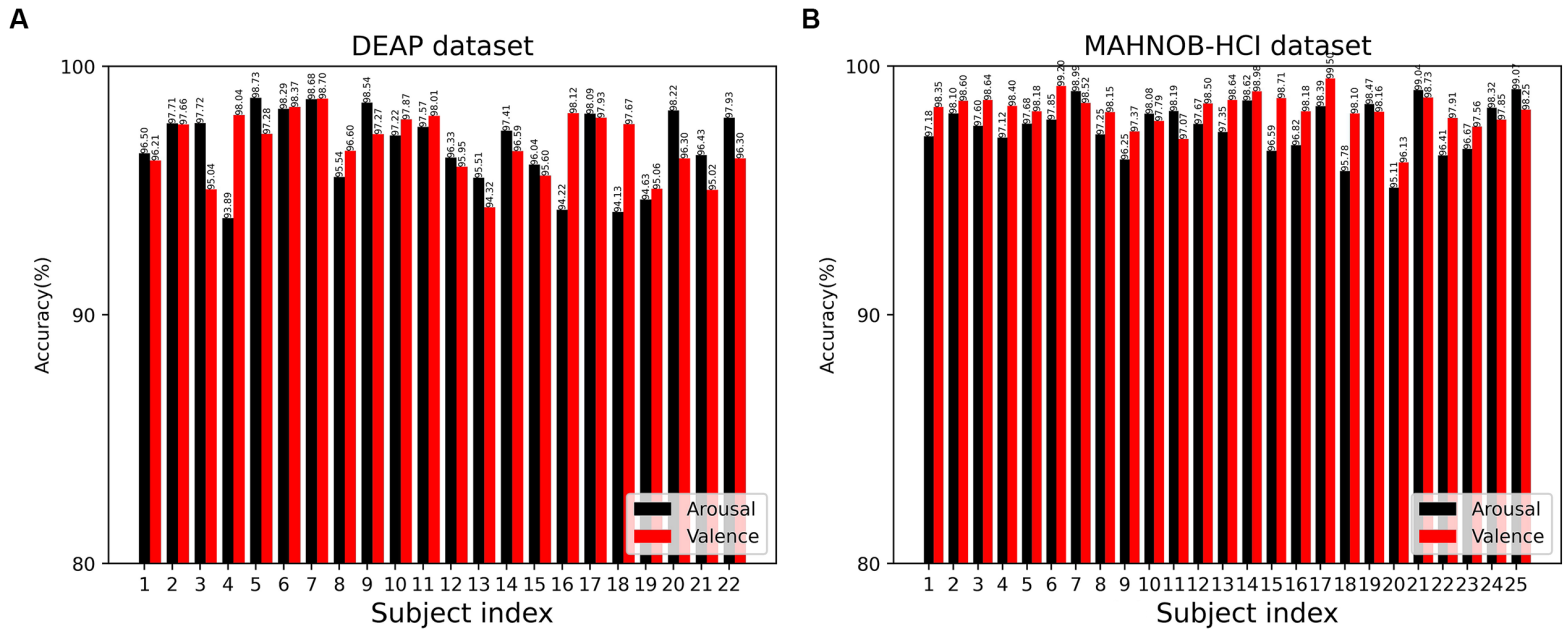
Recognition accuracy of the 3FACRNN network on the DEAP dataset and MAHNOB-HCI dataset for all subjects: **(A)** DEAP dataset; **(B)** MAHOB-HCI dataset.

**Figure 6:**
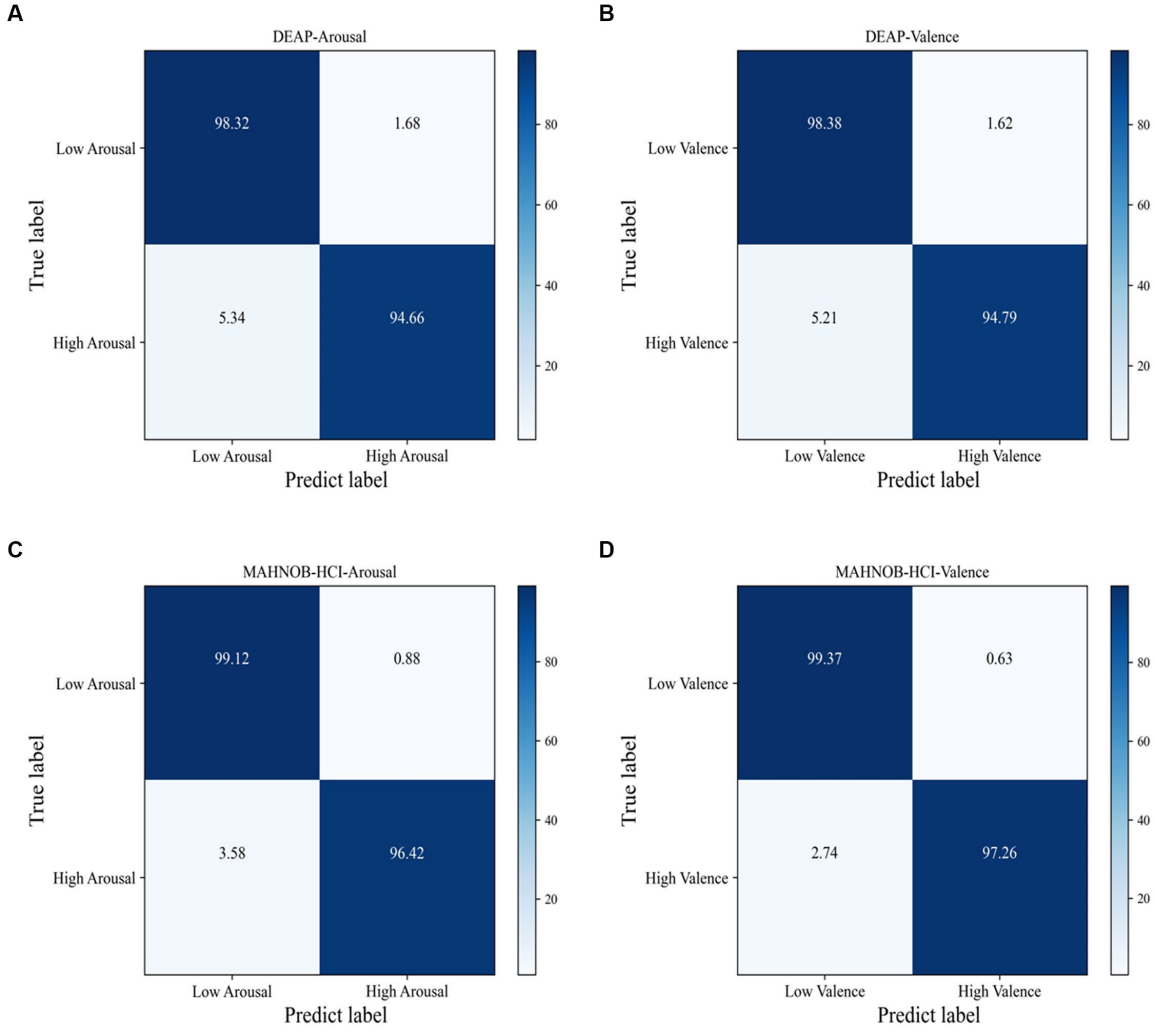
Confusion matrix of the proposed 3FACRNN on DEAP dataset and MAHNOBHCI dataset: **(A)** DEAP:Arousal; **(B)** DEAP:Valence; **(C)** MAHNOBHCI:Arousal; **(D)** MAHNOBHCI:Valence.

The 3FACRNN network produced the greatest recognition results when the grid search parameter w = 1.0. The average recognition accuracy and standard deviation of the 3FACRNN network for all subjects in the DEAP dataset were 96.75 ± 1.75 and 96.86 ± 1.33. This is marginally inferior to the performance on the MAHNOB-HCI dataset, and it is possible that this is due to the fact that the mood induction situation varied between subjects. In addition, for a comprehensive evaluation of the performance of the 3FACRNN network, the F1 Score, a reconciled average of precision and recall, is used as the network model evaluation result. Table 2 demonstrates that the F1 scores of the 3FACRNN network on the emotion and arousal dimensions of the two datasets are 96.09, 96.34, 97.38, and 97.33, respectively. All F1 Scores are greater than 96%, indicating that the 3FACRNN network achieves satisfactory classification results on both datasets. Figure 5 present the accuracies for all subjects in the DEAP dataset and the MAHNOB-HCI dataset, revealing that the 3FACRNN network can achieve more accurate classification results for all subjects in the two datasets, thereby demonstrating its superiority on the two datasets. Figure 6 depict the confusion matrices derived for the 3FACRNN network on the DEAP dataset and the MAHNOB-HCI dataset. Figure 6 demonstrate that the recognition rate of the 3FACRNN network is greater for low valence and low arousal samples than for high valence and high arousal samples, and that the 3FACRNN network can achieve the optimal classification of the emotions for low valence and low arousal samples.

**Table 2:** Accuracy, precision, recall and F1 score obtained by 3FACRNN network on DEAP dataset and MAHNOB-HCI dataset.

Dataset	DEAP		MAHNOB-HCI	
Index	Arousal	Valence	Arousal	Valence
Accuracy	96.75%	96.86%	97.55%	98.37%
Precision	97.57%	97.95%	98.89%	99.13%
Recall	94.66%	94.79%	96.42%	97.26%
F1 score	96.09%	96.34%	97.38%	97.33%

### 3.3 Ablation experiment

In this paper, we validate the effects of multiple attentional mechanisms in the 3FACRNN network model on an emotion recognition task and design four models to compare their performance in an emotion EEG recognition task: the first model contains both the banded attention module and the self-attention module, the second model contains only the banded attention module, the third model contains only the self-attention module, and the fourth model does not contain any attention module. The purpose of constructing these four models was to verify the validity of the self-attention module and the frequency band attention module. Table 3 displays the mean accuracy and standard deviation for each of the four models.

**Table 3:** Average accuracy and standard deviation obtained by the 3FACRNN network for different attention situations.

Attention	DEAP		MAHNOB-HCI	
	Arousal	Valence	Arousal	Valence
With all attention	96.75 ± 1.75%	96.86 ± 1.33%	97.55 ± 1.51%	98.37 ± 1.07%
With only Frequency Band-attention	92.61 ± 3.91%	93.59 ± 3.55%	93.61 ± 3.91%	93.59 ± 3.65%
With only Self-attention	94.84 ± 2.61%	94.35 ± 2.89%	94.22 ± 0.2.37%	94.15 ± 2.46%
W/O any attention	90.11 ± 3.58%	89.43 ± 4.49%	88.53 ± 3.74%	91.18 ± 4.66%

To investigate the effect of various attention modules on the performance of the 3FACRNN network, we compare and analyze the mean accuracy of each network model in Table 3. The first network model, which includes all attention modules, has the greatest improvement in recognition accuracy compared to the fourth network model on the arousal and valence dimensions of the DEAP and MAHNOB-HCI datasets, with improvements of 6.64%, 7.43%, 9.02%, and 7.19%, respectively. With the addition of the self-attention module, the average accuracy of the first network model increased by 1.91%, 2.51%, 3.33%, and 4.22% when compared to the third model. The recognition performance of the network model with the addition of the self-attention module alone is superior to that of the network model with the addition of the frequency-band attention module alone. This is because the frequency-band attention module captures the feature information of different frequency bands in the EEG samples from the local time-slice domain, while The self-attention module captures the intrinsic attentional information between samples.

In order to determine the significance of the 3D feature construction module in the 3FACRNN network model, we input the raw EEG signals directly into the spatial–temporal convolutional network to extract the local spatial–temporal features, bypassing the 3D feature construction module. Because the differential entropy characteristics of the frequency bands in the EEG signal are not utilized in this procedure, the frequency band attention module in the 3FACRNN network is also eliminated, while the other inherent network structures are preserved. First, the raw EEG signals are maintained, then each sampling point is projected onto a spatial matrix, and lastly, the signal matrix is fed directly into a spatial–temporal convolutional network for feature extraction and classification. The average accuracy and standard deviation of the two datasets for the valence and arousal dimensions are displayed in Table 4. Table 4 reveals that the average accuracy and standard deviation of the network model on the two datasets are 94.22 ± 3.12, 93.80 ± 2.69, 93.99 ± 2.57, and 93.74 ± 2.32, with the raw EEG signals as inputs, and that it is lower than the average accuracy of the 3FACRNN network model by 2.53%, 3.06%, 3.56%, and 4.63%, respectively. Experiments have shown that adding the 3D feature construction module to the 3FACRNN network can improve recognition accuracy. This is because the EEG signals processed by the 3D feature construction module are more complex at the feature level and contain more useful emotional information.

**Table 4:** Comparison of recognition performance of 3FACRNN networks with raw EEG signal as input and processed by 3D constructor module as input.

Input EEG signals	DEAP		MAHNOB-HCI	
	Arousal	Valence	Arousal	Valence
With 3D-feature structure	96.75 ± 1.75%	96.86 ± 1.33%	97.55 ± 1.51%	98.37 ± 1.07%
With raw EEG signals	94.22 ± 3.12%	93.80 ± 2.69%	93.99 ± 2.57%	93.74 ± 2.32%

To investigate the effect of visual modalities on the recognition accuracy of the network, we conducted experiments without including visual modalities. Table 5 shows the average accuracy and standard deviation of the network without and with visual modalities. In Table 5, the average accuracy with standard deviation of the network on the two datasets without considering visual modality is 93.47 ± 3.01, 93.18 ± 3.83, 92.46 ± 3.36, and 94.54 ± 3.02, and it is lower than that of the 3FACRNN network considering visual modality by 3.28%, 3.68%, 5.09%, and 3.83%, respectively. This due to the fact that multimodality captures more comprehensive feature information in the global time domain than unimodality, and experimental results show that allowing the EEG modality to learn the dark knowledge of the visual modality improves the recognition performance of the 3FACRNN network.

**Table 5:** Comparison of recognition performance of 3FACRNN networks with and without visual pattern involvement.

Visual feature	DEAP		MAHNOB-HCI	
	Arousal	Valence	Arousal	Valence
With visual feature	96.75 ± 1.75%	96.86 ± 1.33%	97.55 ± 1.51%	98.37 ± 1.07%
W/O visual feature	93.47 ± 3.01%	93.18 ± 3.83%	92.46 ± 3.36%	94.54 ± 3.02%

### 3.4 Analysis of the attention weighting of the frequency average band

**Figure 7:**
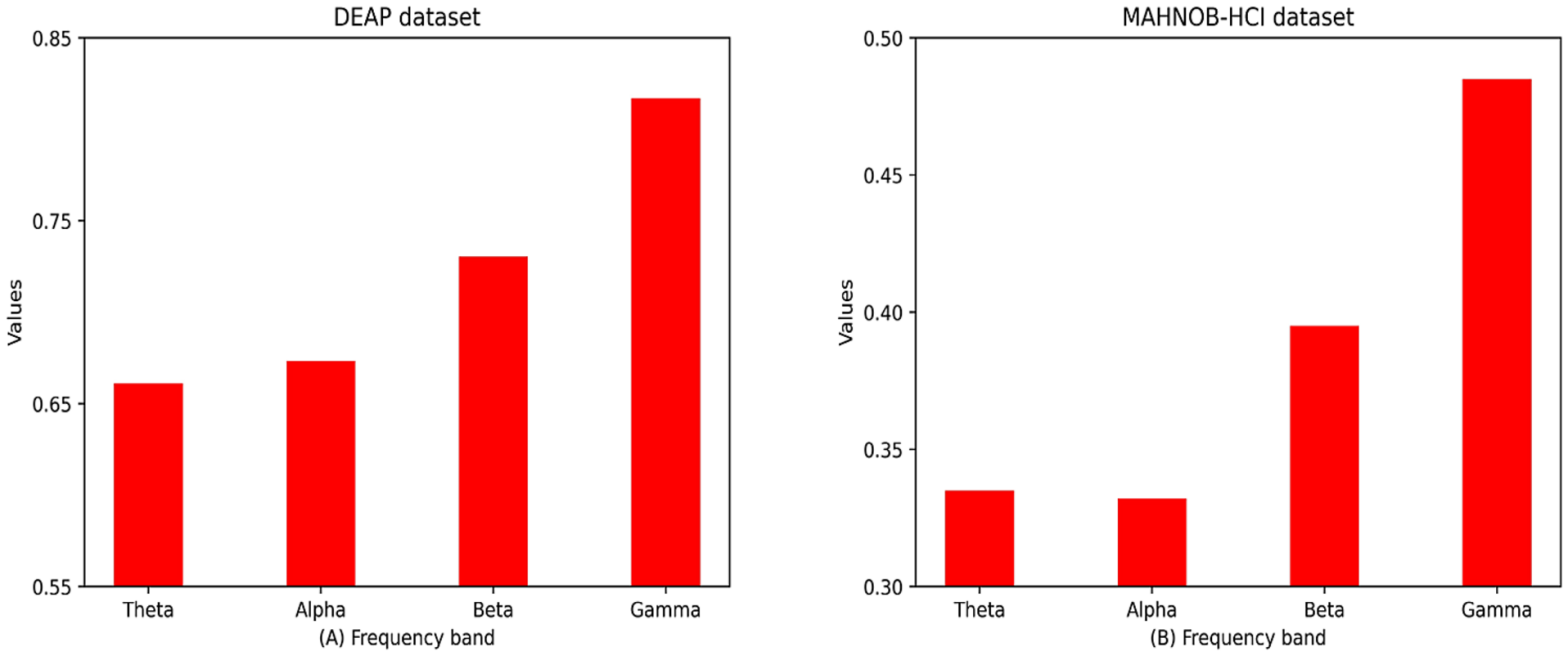
Mean spectral attention masks for all subjects in the DEAP and MAHNOB-HCI datasets in four frequency bands (i.e., theta, alpha, beta, and gamma frequency bands):**(A)** DEAP dataset; **(B)** MAHOB-HCI dataset.

To comprehend the significance of different frequency bands in the emotion recognition process, we calculated the average frequency band attentional weight values of all subjects after training, which represents the significance of different frequency bands in the network training process, and plotted the average frequency band weight rectangles of the four frequency bands in Figure 7. Because the attention weights for each frequency band have been normalized, Figure 7 displays values in the range [0,1] for the attention weights for the four frequency bands. In the DEAP and MAHNOB-HCI datasets, the network assigned the highest attentional weights to the gamma band. Since the network continuously updates the band attentional weights during training, this also suggests that the differential entropy feature of the gamma band provides a more discriminative feature during emotion recognition. Due to the shorter EEG sample duration of subjects in the MAHNOB-HCI dataset, the average band attentional weights of the four bands are lower in the MAHNOB-HCI dataset than in the DEAP dataset.

### 3.5 Method comparison

We compared the proposed 3FACRNN network model with the state-of-the-art methods on the DEAP dataset and MAHNOB-HCI dataset, as shown in Table 6, with a brief description of each method as follows:

1. DBN (Wang and Shang, 2013): A Deep Belief Network (DBN)-based emotion recognition system that automatically extracts features from four channels of raw EEG data in an unsupervised manner and accomplishes emotion classification.
2. M-CLASS (Sander and Ioannis, 2013): A Multimodal Emotion Recognition Method for Emotion Recognition after Fusion of Facial Expression Features and EEG Features at Decision Layer or Feature Layer.
3. Conti-CNN (Yang et al., 2018b): A three-dimensional input continuous convolutional neural network combining features from multiple bands to improve the accuracy of emotional EEG recognition.
4. CRAM (Zhang et al., 2019): An emotion recognition network that uses CNNs to abstractly encode EEGs and a recursive attention mechanism to extract spatial–temporal features in EEGs for emotion classification.
5. GCNN (Song et al., 2020): A network that uses spectrogram filtering to extract different differential entropy features for emotional EEG recognition.
6. CNN-LSTM (Chen et al., 2020): An emotional EEG signal recognition network using a hybrid convolutional recursive module of CNN and LSTM.
7. 4D-CRNN (Shen et al., 2020): A four-dimensional convolutional recurrent neural network is proposed to convert the differential entropy features of different channels into a 3D structure to train the network model.
8. CSDNN (Liu J. et al., 2020): An emotion recognition network combining convolutional neural networks with sparse autoencoders.
9. MDBN (Wang et al., 2020): Multimodal emotion recognition using deep belief networks.
10. DCNN (Gao et al., 2021): A dense convolutional neural network for sentiment recognition using channel fusion methods.
11. SFENet (Deng et al., 2021): An emotion recognition network based on spatial folding integration.
12. GCN-LSTM (Yin et al., 2021): An emotion recognition algorithm based on graph convolutional neural networks and long and short-term memory neural networks.
13. Multi-CNN (Huang et al., 2019):A deep convolutional neural network combining facial expressions and EEG for enhanced emotion recognition.
14. MA-attention (Zhong et al., 2020): A convolutional neural network using moving average (MA) and attentional mechanisms was designed to recognize emotional EEG signals.
15. Deep learning (Siddharth and Jung, 2022): A deep learning method is designed to implement a multimodal vision and EEG based affective computing network using deep learning methods.
16. HC-MFB (Zhang et al., 2022):A multimodal emotion learning network model based on heterogeneous convolutional neural networks and multimodal factorized bilinear pools is designed.
17. CADD-DCCNN (Li et al., 2023): A causal convolutional neural network based on cross-attention mechanism for EEG emotion recognition.
18. TR&CA (Peng et al., 2023): An emotion recognition network based on channel attention mechanism and time relative coding mechanism.

**Table 6:** Comparison of mean accuracy and standard deviation (acc ± std.%) between the baseline method and the proposed 3FACRNN network on the DEAP dataset and the MAHNOB-HCI dataset.

Author	Methods	Year	DEAP		MAHNOB-HCI	
			Arousal	Valence	Arousal	Valence
D. Wang	DBN	2013	60.9	51.2	-	-
K. Sander	M-CLASS	2013	-	-	66.5	71.5
T. F. Song	GCNN	2018	87.72 ± 3.32	88.24 ± 3.18	-	-
Y. Yang	Conti-CNN	2018	81.55 ± 6.55	82.77 ± 4.47	-	-
D. Zhang	CRAM	2019	84.46 ± 9.27	87.09 ± 7.49	-	-
Y. G. Huang	Multi-CNN	2019	-	-	74.17	75.21
J. Chen	CNN-LSTM	2020	93.26	93.64	-	-
F. Shen	4D-CRNN	2020	94.58 ± 3.69	94.22 ± 2.61	-	-
J. Liu	CSDNN	2020	92.86	89.49	-	-
Z. Wang	MDBN	2020	87.32	83.69	-	-
X. L. Zhong	MA-attention	2020	-	-	70.25	73.27
Z. Gao	DCNN	2021	92.92	92.24	-	-
X. Deng	SFENet	2021	91.94	92.49	-	-
Y. Yin	GCN-LSTM	2021	90.60	90.45	-	-
Siddharth	Deep learning	2022	-	-	80.42	80.77
Yong Zhang	HC-MFB	2022	-	-	90.37	90.50
C. Li	CADD-DCCNN	2023	92.42	90.97	-	-
G. Q. Peng	TR&CA	2023	95.58 ± 2.28	95.18 ± 2.46	-	-
Ours proposed	3FACRNN	2023	96.75 ± 1.75	96.86 ± 1.33	97.55 ± 1.51	98.37 ± 1.07

Table 6 reports the comparison of the recognition performance of all the above methods and the proposed 3FACRNN network in this paper on DEAP and MAHNOB-HCI datasets. Overall, the proposed 3FACRNN network outperforms the state-of-the-art methods with average recognition accuracies of 96.75, 96.86, 97.55, and 98.37 on DEAP and MAHNOB-HCI datasets, respectively, with standard deviations of 1.75, 1.33, 1.51, and 1.07, respectively.

We compared the 3FACRNN network with the Conti-CNN method proposed by Yang et al. The 3FACRNN network outperforms the Conti-CNN method in average recognition accuracy by 15.2% and 14.09%, respectively, which is due to the fact that Yang et al. only considered the feature information of the EEG signals in terms of spatial domain, and did not take into account the EEG signals in terms of time domain feature cues, while the 3FACRNN network utilizes the LSTM network to obtain the long-term temporal features of emotional EEG signals, which improves the network’s prediction performance of emotional states.

Although Chen et al. used a hybrid CNN and LSTM convolutional recurrent neural network to extract the feature information of EEG signals in both spatial and temporal domains, the average recognition accuracy was still 3.49% and 3.22% lower than that of the 3FACRNN network, which is due to the fact that the 3FACRNN network not only uses a convolutional recurrent neural network based on CNN and LSTM to extract the spatial and temporal feature information of the EEG signals but also incorporates a frequency band attention module and a self-attention module to enhance the discriminative property of the feature information. In addition, Zhang et al. incorporated a recursive attention mechanism into a convolutional neural network to explore the effect of different time-slice samples on the emotion recognition process, but their average recognition accuracy was lower than that of the method proposed in this paper, which further illustrates the effectiveness and advanced nature of the multi-attention mechanism chosen in this paper.

Yin et al. used EEG signals obtained from different electrode channels to construct a brain network, and adopted the brain network representation learning method of graph neural network to obtain the feature representation of EEG signals in spatial and temporal dimensions, and finally extracted the temporal features of emotional responses using LSTM network, which can achieve an average recognition accuracy of 90.60% and 90.45%, but since graph neural network needs to perform the feature vector computation while adjusting the structure between brain network graphs, so the efficiency of the algorithm will show a significant decrease with the increase of feature graphs. The 3FACRNN network spatially projected the EEG features in order to maintain the relative relationship between the placement of EEG electrodes on the head. The EEG features of each frequency band were first mapped into a 2D matrix and then organized into a 3D structure according to the frequency bands, the 3FACRNN network outperformed the GCN-LSTM method of Yin et al. by 6.15 and 6.41%, respectively, in terms of average recognition accuracy, and also outperformed the GCN-LSTM method in terms of algorithmic efficiency.

Siddharth et al. used deep convolutional network to extract the emotional feature information of visual modality and EEG modality and combined the two feature information for the prediction of emotional state with an average recognition accuracy of 74.17% and 75.21%. Zhang et al. proposed a multimodal emotion learning network model based on heterogeneous convolutional neural network and multimodal factorized bilinear pool. The network model fuses the feature information of visual modality and EEG modality in the decision layer, and the average recognition accuracy can reach 90.37% and 90.50%. In this paper, we propose a multimodal emotion recognition network, 3FACRNN, which utilizes a multitask loss function Lc to force approximation of intermediate feature vectors of visual and EEG modalities in order to improve the recognition performance of the EEG network through visual knowledge. The 3FACRNN network takes into account the differences between different modal features, and its average recognition accuracy is higher than that of the Zhang et al. and Siddharth et al. proposed methods.

## 4 Discussion

In this paper, we conducted extensive experiments using 3FACRNN networks on two public datasets and obtained satisfactory performance, and we next discuss points in the 3FACRNN networks that can be further refined.

We add a 3D feature construction module to the 3FACRNN network, which projects the electrode position information of the EEG samples into a 2D matrix to facilitate the subsequent convolution operation. We set the length and width of the 2D matrix to 9×9. We designed a more compact 2D matrix compared to the sparse matrix used by Li et al. The compact matrix has a smaller size and requires relatively fewer convolutional kernels, consuming less time cost, while the sparse matrix requires more convolutional kernels to extract more features from the EEG samples, which is more favorable for the subsequent recognition classification task. So next we will investigate the application of sparse maps in the field of multimodal EEG sentiment recognition.

From Table 6, we can see that the 3FACRNN network outperforms the CRAM method in both dimensions of the DEAP dataset, producing a significant increase of 12%, which can be attributed to the fact that the convolutional recurrent neural network of the 3FACRNN network is much deeper, containing four convolutional layers, one pooling layer, one linear layer and one LSTM layer, and producing a feature map with {64,128,256,128} feature maps, whereas the CRAM method contains only one convolutional layer, one pooling layer and one LSTM layer, producing {40} feature maps, and its network depth is much lower than that of the 3FACRNN network. Deeper convolutional and pooling layers also allow the 3FACRNN network to extract and retain more emotion-related cues.

## 5 Conclusion

In this paper, we propose a 3FACRNN network model for multimodal emotion recognition, which includes two parts, the EEG network and the visual network. A 3D feature construction module was added to the EEG network with the aim of complexly extracting the electrode information, frequency band information and spatial–temporal information from the original EEG signal to provide more feature cues to the convolutional recurrent network; In addition, we used the frequency band attention module and the self-attention module to make the feature information extracted by the convolutional recurrent network more discriminative at both local and global time slice scales. Finally, the two network models reduce the proximity of the intermediate feature maps through a multi-task loss function L_C_, which allows the EEG network to learn the knowledge of the already trained visual network and improves the performance of the EEG network for affective computing. We pre-train the visual models using CNN and TCN, and then use the spatial–temporal features in the trained visual networks as dark knowledge to improve the recognition performance of EEG networks. The experimental results demonstrate the effectiveness of our proposed 3FACRNN network model. The 3FACRNN network can understand the feature information of different modalities, and it can also complexify the feature information through the 3D feature construction module and the multi-attention mechanism, so as to make it contain more information conducive to the recognition of emotions, and to improve the recognition performance of the network. In future work, we will apply the 3FACRNN network to topic-independent and cross-session tasks to improve the generalization of the model.

## Data availability statement

The original contributions presented in the study are included in the article/supplementary material, further inquiries can be directed to the corresponding author.

## Ethics statement

Written informed consent was obtained from the individual(s) for the publication of any potentially identifiable images or data included in this article.

## Author contributions

YD: Data curation, Methodology, Software, Writing – original draft, Conceptualization, Investigation, Visualization. PL: Conceptualization, Data curation, Investigation, Methodology, Software, Validation, Writing – review & editing. LC: Investigation, Methodology, Validation, Writing – review & editing, Conceptualization. XZ: Supervision, Writing – review & editing, Formal Analysis. ML: Investigation, Validation, Writing – review & editing. FL: Investigation, Supervision, Writing – review & editing.
